# Discussion on Anæsthetics in the New York Academy of Medicine

**Published:** 1860-04

**Authors:** 


### ARTICLE XIV.

### *Discussion on Anæsthetics in the New York Academy of Medicine.*

At the meeting of December 21st, an interesting discussion on the use of anæsthetics was incidentally started, and kept up nearly until adjournment.

Dr. Kissam related his experience with the two principal anæsthetics, sulphuric ether and chloroform, as employed in surgery; he gave preference to chloroform, but recommended it to be used with great caution.

Dr. Reese coincided with Dr. Kissam. As to the different action which the two articles possess, he would state that he was in the habit of using chloroform in puerperal convulsions, with good effect, while ether could not be used in this disease, because it rather increased, if anything, the tendency to convulsions. In reference to chloroform, he had noticed that the Paris correspondent of the *New York Times*, who was a physician, had stated that the use of this remedy had been lately attended with exceedingly fatal results in the Parisian hospitals. It was Trosseau, he thought, who had introduced and sanctioned the administration of chloroform by an apparatus in which the inhalation is made through one nostril—the other nostril being left open for the reception of atmospheric air. Great stress is laid upon the fact, that chloroform, as thus administered through the open nostril, and in such a way as to admit the atmosphere, prevents any injurious results from the accumulative action of the remedy, which are so often alarming. He had never in his own hands had a case in which the chloroform was attended by a fatal result, or even approached to fatality. He always introduced the remedy very gradually, withdrawing it occasionally, and never carrying it so far as to produce profound stertor or coma, which in no case, where the anæsthetic was resorted to, he deemed it necessary to produce.

Dr. Squib had used chloroform for the past five or six years in many cases, perhaps as often as a hundred times, for various affections, and had never seen it followed by disastrous results. He is not in the habit of giving it very profusely, very often giving hardly enough to deprive the patient of sensibility or ordinary sensation. Used in this manner, it has proved a safe agent in his hands. He had given it as long as 45—50 minutes at a time, without producing any dangerous effects. The danger from its use he considers to arise chiefly from the rash administration of it, being given in many cases so as to prevent the admission of air. When mixed with air it is certainly far more safe than when that due admixture of air is not given.

The President, Dr. Watson, stated that he would like to have the distinction pointed out between the results of the use of ether and of chloroform.

Dr. Minor: During eight years experience in the administration of anæsthetics, in the hospital to which he was attached, the free admission of atmospheric air with the anæsthetic agent has always been secured, and he had never yet met with more than one case, which appeared in his opinion to be dangerous. In that instance there was suspended animation for five or ten minutes; the patient, however, finally recovered. As a general rule, the agent is administered according to the plan laid down by Dr. Metcalfe, some years ago, in a paper which was read before the Academy. In some cases Dr. M. had seen chloroform administered, by young gentlemen, rather in a careless manner; as they become interested in the operation, they are apt to apply the sponge too closely to the patient's nostrils, and thus prevent, to a great degree, the admixture of air with the vapor of the anæsthetic. He believed that where chloroform is given with due care, and the quantity regulated according to the peculiarities of the case, there will be comparatively little danger following its use; when, on the other hand, it is carelessly or ignorantly administered, it is a dangerous remedy, and is not unfrequently followed by fatal results ; in fact, he believed that most of the fatal cases can be traced to a careless administration of the remedy. These remarks, of course, referred to chloroform alone. In many instances he had used a mixture of chloroform and ether, the pecentage of the latter, however, being so small, that as far as the ultimate effects were concerned, it might be considered as chloroform.

Dr. Squib thought that in many instances a second rate article of chloroform was used. Many gentlemen are in the habit of using a sample imported from England, under the impression that they are using the article prepared by alcohol ; while it is in fact that prepared by amylated ether, neither as pure nor as efficient as that prepared by alcohol.

President: I hope the gentleman does not mean to say that we use an impure article, and in this way produce unpleasant results?

Dr. Squib: I think that statistics show this to be the case.

Dr. Gardner stated that he had used chloroform chiefly, and that his experience with ether was limited. He thought that he gave chloroform in the first case of obstetrics in which it was given in New York. He had given it so far without the least hesitation. He had used chloroform principally because it supplied a want which ether failed to supply altogether. In several cases, even a pound of ether failed to produce the desired effect, which would result from only a tea spoonful of chloroform. Again, it is very generally admitted by accouchers that ether is inadmissible in cases of puerperal convulsions, which are aggravated by its use. Whenever he deemed the use of an anæsthetic advisable, he generally had resorted to chloroform, and he would remark, that even where patients are supposed to be particularly obnoxious to its use, as in organic diseases of the heart and lungs, it may be resorted to in obstetric practice with benefit, instead of leaving the patients to the injuries which may be caused by the natural efforts of a difficult and protracted labor; the danger from chloroform was, in his opinion, far less than the injury resulting from the straining efforts in these cases. Under the administration of chloroform these are mitigated; labor is carried on by the involuntary muscles principally, and as a consequence, the tax upon the heart is far less. He had great confidence in the use of chloroform in those ordinary cases of labor, where, although the patient has a large, roomy pelvis, dilated and moist vagina, and there is no reason why the labor should not terminate in a short time, the os remains rigid, and this rigidity continues for hours without apparent change, and in spite of the most severe expulsive efforts of the uterus. The patient is worn out by these efforts, and rupture of the uterus or other injuries threaten. Now, the inhalation of a tea spoonful or two of chloroform, in these cases, seldom fails to ease the patient, relax the os, and allow the speedy expulsion of the fœtus. In cases of difficult labor, with unrelaxed and rigid os, it had been his custom to resort to chloroform instead of bloodletting, opium, tartar emetic, or the warm douche, which latter he considered the next best thing. As a general rule, the *cervix of the uterus* is readily brought under the relaxing influence of the agent, while it often requires a considerably larger quantity to relax the muscular fibres *of the body of the organ*, which is very desirable in cases where the operation of turning has to be performed. Dr. Gardner remembered one case, in which he was called to attend, in company with another gentleman, where version was required; it being an arm-presentation. Chloroform was administered, and the gentleman in attendance was endeavoring to find the feet. After some fifteen minutes or half an hour spent fruitlessly, he gave it up, stating that he had reached the fundus of the uterus, but could not find the feet, and requested Dr. G. to examine the case. On introducing the hand, Dr. G. found that what the former had mistaken for the fundus, was not in reality the fundus, but the circular fibres of the organ, which were firmly contracted around the child. The feet of course were above this point, surrounded by this ring of muscular fibre, and entirely out of reach. On recognizing this condition of things, the woman was placed under the full influence of the anæsthetic. This had the momentary influence of relaxing the fibres, during which time the hand was passed through, the feet seized, and turning performed. By this means, then, we are able to perform one of the most formidable operations in midwifery with comparative ease and comfort. So far as the application of the forceps is concerned, he was not in the habit of applying them, with the patient under the full influence of chloroform ; he preferred that the patient should be sensible, so that if the instrument should press against the soft parts, in a manner that may be injurious to the woman, he might be made aware of it by her movements.

Dr. Peaslee remarked that in regard to the danger accompanying the administration of chloroform, a distinction must be made between its use in obstetric and in surgical practice. In the former it is comparatively safe, because the paroxysms and labor pains react, to a certain extent, against the influence of the anæsthetic, while it is much more fatal in surgical practice. In regard to the two agents, he preferred ether ; he had commenced with it, and used it up to the present time. In the first few trials, unpleasant effects were occasionally seen, which Dr. Peaslee now feels certain to have been due to the manner in which it was administered. His own experience coincided with the results of Dr. Snow's experiments, and convinced him that the safety of the administration of this agent, depends *cœteris paribus*, upon the proper amount of atmospheric air being given with it. With this precaution, Dr. P. has never seen it followed by any unpleasant consequences whatever, and furthermore, he had never known, seen, read, or heard of a case, where it was followed by unpleasant effects for more than a few hours. In some cases, he had given as much as two pounds of ether, and not unfrequently one pound. Chloroform he used with the utmost care, and took the precaution, mentioned by Dr. Snow, never to give it for more than twenty minutes at a time. In one case, however, even with these precautions, respiration was interrupted for three or four minutes, and for a time he thought that the patient was dead, and it was only by establishing artificial respiration that recovery finally took place. In obstetric practice, where there is a rigid os, chloroform has a decided influence in relaxing the muscular fibres which constitute a sort of sphincter muscle at this point; it appears to exert an almost specific influence on that portion of the uterus, almost as certainly and successfully as belladonna acts upon the eye.

Dr. McNulty thought that in considering this subject, the first question should be, what is the exact cause of death? This being known, it could readily be ascertained which of the two anæsthetics, ether or chloroform, contains the most dangerous properties. The manner in which death is produced by these agents seemed to him to be the first subject of inquiry.

The President, Dr. Watson, inquired of Dr. Peaslee, whether in the obstetric use of these remedies, hemorrhage after delivery is apt to be increased thereby.

Dr. Peaslee thought that this was decidedly the case, when chloroform is first given, there is for a short time a diminution in the power of the uterine contractions. He had noticed that on removing the placenta there was considerable tendency to hemorrhage in these cases. To counteract this, he was in the habit of giving a dose of ergot, perhaps thirty drops of the tincture ten or fifteen minutes before the delivery. Taking that precaution he had never seen any dangerous result.

Dr. H. Green had never employed ether; chloroform he had used for ten years, and with one exceptional case, which did not, however, prove fatal, had never seen any difficulty or danger from its administration. In obstetric cases he had noticed one fact, which he had not yet heard mentioned, namely, that after its administration the necessity of giving laxatives on the second or third day was obviated, the anæsthetic having a tendency to relax the bowels.

Dr. Raphael stated that in his practice he had used chloroform invariably, and had never seen it attended with injurious results, except in one instance, which occurred in a boy between fifteen and sixteen years of age, from whose neck a tumor was removed. The boy remained insensible for nearly three-quarters of an hour, but finally recovered. He believed that death was sometimes produced by a careless administration of the agent, especially when it is poured upon a sponge and placed directly over the nostrils. In this case, apart from the prevention of a free access of air, we have the irritating action of the chloroform producing a spasm of the glottis, which in some cases is, perhaps, so severe as to produce death.

[This would seem improbable as the local effect of chloroform upon muscular fibre is not to produce *contraction*, but, as Dr. Faure has shown by his experiments, when it is brought in contact with muscular tissues, it destroys instantaneously their contractibility. Taking this in connection with the facts so ably presented in the subsequent remarks of Professor Dalton, there can hardly remain a doubt, that chloroform produces death by paralysis of the cardiac muscle, whenever it circulates in sufficient quantity in the blood to exercise this, its *peculiar*, paralysing influence. Can this be avoided in every case? Not until we are able to determine the absorptive power in every individual, to measure in every particular case the resistance of the muscular fibre to this paralysing effect, which undoubtedly varies in various persons, according to sex, age and idiosyncracy; until we know all this beforehand, we will be no more able to foretell its effect, than we are, to predict, whether a certain dose of mercurial will produce ptyalism or not.—GOTHAM.]

Dr. O'Reily related four cases in which chloroform was administered, in all of which the most alarming symptoms took place suddenly, although the agent had been administered with care. By most energetic means, however, they were all restored.

Dr. Dalton made the following remarks:

Of course, Mr. President, I have very little experience with regard to the effects of these two agents upon the human subject, although I had the pleasure of witnessing the first operation in which ether was used as an anæsthetic agent. In my own practice, if you may call it such, the patients have been principally animals. I presume, however, that there is very little difference in their mode of operation on animals and on men. When I commenced, I, of course, used ether; but as ether requires to be given in a very large bulk, I soon found it very inconvenient, and commenced using chloroform in its stead, and found it very much more pleasant for myself, because it was more easily administered to the animals, and I continued to use it for a certain time. Very soon, however, I found that the animals would occasionally die, which I attributed to some imperfection in the mode of administering the agent. I continued the practice, but still the accident referred to would occasionally occur. Not to take up too much time in detail, the simple fact is, that, at the end of six months, from the time I commenced its administration, I abandoned it. Sometime afterward I again had occasion to use it; I gave it, but found that it was followed by the same results. Since that time I have given it up altogether, and instead of it I have used sulphuric ether. I think I may say, without exaggeration, that I am thoroughly convinced that there is a radical difference in the danger following the administration of these two substances. I am sure that chloroform is more dangerous to animals, at least; whether it is so in man or not, I do not know.

In order to understand this subject thoroughly, it is necessary that we should endeavor to ascertain the manner in which death results in the fatal cases. Death sometimes follows without any evident or traceable cause. It may occur from ether or chloroform by a very careless administration, or from an impurity of the article, provided that the patient breathes nothing but the vapor of the ether or chloroform. Now in these cases, death is not attributable to the ether or chloroform ; it is simply due to the want of atmospheric air. If you give a man a grain of opium and then stop his mouth and nostrils, he will of course die ; but certainly not from the opium, but from the want of atmospheric air. The same is true of the administration of ether or chloroform. Therefore, the first thing to be attended to, when we wish to prevent a fatal issue from the administration of these substances is to see that they are mixed with a sufficient quantity of atmospheric air, and then one cause of death would be excluded.

Sometimes, however, even with all our precaution, we find the respiration and the heart stopping suddenly and the patient dead. It is an interesting question to know whether or not death is produced by the *stoppage of respiration* or *of the heart*. My own belief is, that in the case of chloroform, death is produced by paralysis of the heart. My reasons for this view are two-fold.

In the first place, if you moderately etherize or chloroformize an animal, carrying it carefully just up to the point of insensibility, and then open the walls of the chest as quickly as possible, the lungs will of course, collapse, and respiration be at an end, but the heart will continue to beat for a considerable length of time. If, on the other hand, you etherize or chloroformize an animal until respiration is stopped, and then open the chest, you will find the heart still beating, but very feebly. I have several times performed the following experiment, namely: to etherize an animal moderately, but enough to deprive it of all sensibility, then immediately the chest was opened and the animal laid aside; another animal was then etherized until death was produced, and on immediately opening the chest the heart was found still, while in the first animal it was yet beating. So far as this goes, it tends to show, with a great deal of conclusiveness, that the fatal result is produced by a direct paralysis of the heart.

In experimenting thus with animals, I have had occasion to notice very frequently, when the anæsthetic is carried only to the stoppage of respiration, that the animals usually recover, and expect with confidence that respiration will begin again; but if, on noticing that the respiration is stopped, I find the heart itself still, I know that the animal is dead, although I have noticed, after the circulation is at an end, that it is sometimes re-established in a certain manner which is entirely characteristic, and being once seen, is very readily recognized. This is, however, entirely unavailing; the animal never recovers.

In my own experience, then, fatal results have followed both ether and chloroform. I have killed dogs and cats with ether and chloroform, but I am obliged to take a great deal of pains to produce this result with ether, whereas death often follows the use of chloroform, notwithstanding the best precautions. It has been said, that when death occurs from the administration of chloroform in the human subject, that it is attributable to giving it too rapidly or too abundantly; but while there are undoubtedly many cases in which injurious results follow from the non-admission of a sufficient amount of air, still I am of the opinion that the injurious or fatal results cannot be always attributed to that cause, for the reason that these accidents have occurred in the practice of our best and most careful surgeons, who invariably exhibit this remedy with the utmost caution, and yet, when every thing appears to be going on well, the patient suddenly dies. So far, we know of no precaution which will prevent the occasional occurrence of this accident.

President: I would inquire of Dr. Dalton whether the effects of these agents are cumulative?

Dr. Dalton: I cannot say that they are. Anæsthetics taken in by the lungs enter the blood so very quickly that I should not think there would be any cumulative effect.

President: In some cases the patient seems to recover so as to speak, and yet in a few moments dies. Did you ever see anything of that kind in animals?

Dr. Dalton: The only thing I have seen analogous to that, is the spasmodic respiration after the stoppage of the circulation, which led me to believe, when I first saw it, that the animal would recover, I now know, however, that these efforts are entirely unavailing. When once the heart has ceased to pulsate, the animal is dead.

Dr. O'Reily gave his theory of the fatal action of chloroform. In his opinion, the anæsthetic acted primarily upon the ganglionic nervous system, by which the proper process of endosmose and exosmose in the lungs is disturbed, and the air cells in the lungs are prevented from duly decarbonizing the blood.

Dr. Buck related the history of the death from chloroform which occurred several years ago at the New York Hospital. The patient in that case was a perfectly healthy man. Two weeks before the unfortunate accident occurred he had taken chloroform, which produced no disagreeable result whatever, neither were any greater precautions taken at this time than when the fatal issue occurred. In his case there was nothing whatever to forbid the use of this agent. Neither can the unfortunate result be attributed to the impurity of the chloroform, for the same article had been administered a day before to one of the patients, with the very best effect. In this instance the quantity was by no means large, certainly less than a drachm. The accident occurred very suddenly indeed. Dr. B. had barely time to order the assistant to bring a wet cloth, and then felt of his pulse, but it was gone. He did not know how any greater precautions could have been used than had been in this case.

Dr. Batchelder: had tried experiments with chloroform upon himself frequently, by inhaling it through one nostril while the other was stopped, and then watching the effects closely. At first a singing in the head and ears were felt, then flashings of light before the eyes, assuming various shapes, were seen, and a prickling sensation was felt in the fingers. If carried beyond this point, it begins to affect the voluntary muscles, so much so that in some instances he would have fallen down if he had not stopped inhaling the agent. In relation to the manner in which it produces death, he agreed entirely with Dr. Dalton's views. He thought that the heart did not become paralyzed until all the voluntary muscles had become so. He had seen but two cases in which the agent produced alarming symptoms. They were both surgical, and were rescued by inducing artificial respiration.

Dr. Gardner regarded the minimum dose of 20 drops, as generally stated in books, as too large. In one case he had seen a sufficient anæsthetic effect produced to allow the painless extraction of a tooth, by the inhalation of only 5 drops of chloroform.

As regards the internal administration of chloroform, reasoning from the effects of ether in this way, he had used it quite largely at the time when cholera was prevalent, but it did not seem to produce any effect at all. Since that time he had given it frequently in various gastric and intestinal diseases, but was unable to observe any beneficial effect whatever.

Dr. McNulty observed that from the turn which the discussion had taken, one would draw the inference that sulphuric ether was a perfectly harmless and safe agent, and could be administered almost *ad libitum*. He certainly had that impression very strongly. Last week, however, he had occasion to administer sulphuric ether to his own brother, and of course all possible precautions were used. He was put in the recumbent posture, and an abundance of atmospheric air was secured with the vapor inhaled. For the first five minutes the proportion of ether to air was certainly not more than 1 in 20, perhaps only 1 in 50. From that time its administration was carried on very gradually, and he was apparently coming under the influence of the anæsthetic very well indeed. Suddenly, when looking at his face, he was struck with its remarkable pallor. On feeling the pulse it was found a mere flutter; the respiration had also ceased. The sponge was immediately taken away, and Marshall Hall's ready method resorted to. The patient gradually came to, but did not fully recover until six hours afterward. This case, the Doctor remarked, has certainly shaken my confidenee in the safety and harmlessness of this remedy very much indeed.

The President, Dr. Watson, inquired of Dr. Dalton whether he had found ether *fail* to produce all the effects that can be wished for from chloroform.

Dr. Dalton: It has never failed in my hands. My experience would lead me to believe that it is capable of producing all the effects of chloroform.

Dr. McFarland remarked that he had been in the habit of using chloroform internally quite largely. His experience was directly contrary to that of Dr. Gardner. In the ordinary cases of flatulentia enterica he had used it with almost uniform success, in many cases relieving the pain in a very few minutes. But now for a more remarkable case, one of *colica pictonum*, which occurred in a painter who had been working in a room which had to be closed on account of the coldness of the weather. The close confinement brought on an attack of colica pictonum, for which Dr. McF. attended him, and carried him through by means of morphia, croton oil, and iodide of potassium. Not long after, he had a second attack, which was very severe. After having given him morphia, croton oil, and iodide of potassium, without producing the desired effect, the Doctor concluded to put him upon the internal use of chloroform. He first gave him 20 drops, which was very soon vomited up. He then gave him ten drops every half hour until he had taken six or seven doses, when all the distressing symptoms disappeared, and strange to say, his bowels became relaxed, and he had copious evacuations. The chloroform was administered for two days. The iodide of potassium was given to counteract the effects of the lead, a few days after the exhibition of which the blue line around the gums disappeared. He was, however, again attacked some time later, having worked as before in a closed room. On this occasion the chloroform treatment was at once resorted to, and it had precisely the same effect. Now, if such a simple remedy as that will relieve a case of colica pictonum in one instance, it is certainly worthy of trial in another. [*Med. and Surg. Reporter.*

